# Understanding the bullying phenomenon through the eyes of the youth football coaches in the Portuguese region of Tâmega e Sousa

**DOI:** 10.3389/fspor.2025.1520737

**Published:** 2025-03-17

**Authors:** Cátia Vaz, José Eduardo Teixeira, Daniel L. Portella, Diogo Monteiro, Pedro Forte, Sandra Silva-Santos, Joana Ribeiro

**Affiliations:** ^1^ CI-ISCE–ISCE Douro, Penafiel, Portugal; ^2^ Education Department, Higher Institute of Educational Sciences of the Douro, Penafiel, Portugal; ^3^ Transdisciplinary Research Center in Education and Development (CITEB), Instituto Politécnico de Bragança, Bragança, Portugal; ^4^ Polytechnic of Guarda, Guarda, Portugal; ^5^ Department of Sports Sciences, Polytechnic Institute of Bragança, Bragança, Portugal; ^6^ Research Center in Sports, Health and Human Development, Covilhã, Portugal; ^7^ SPRINT – Sport Physical Activity and Health Research & Innovation Center, Rio Maior, Portugal; ^8^ Research Centre for Active Living and Wellbeing (LiveWell), Polytechnic Institute of Bragança, Bragança, Portugal; ^9^ Department of Sports Sciences, Polytechnic of Cávado and Ave, Guimarães, Portugal; ^10^ SPRINT – Sport Physical Activity and Health Research & Inovation Center, Guarda, Portugal; ^11^ Group of Study and Research in Physical Exercise Science, University of São Caetano do Sul, São Caetano do Sul, Brazil; ^12^ Master Program in Innovation in Higher Education, University of São Caetano do Sul, São Caetano do Sul, Brazil; ^13^ ESECS-Polytechnic of Leiria, Leiria, Portugal; ^14^ Sport Department, Higher Institute of Educational Sciences of the Douro, Penafiel, Portugal; ^15^ Centre for Research, Development and Innovation – CIDI/IEES, Fafe, Portugal; ^16^ School of Sport and Leisure, Polytechnic Institute of Viana do Castelo, Viana do Castelo, Portugal

**Keywords:** bullying, football, children, youth, sports coach

## Abstract

**Introduction:**

Bullying is a serious social problem affecting, primarily, children and adolescents in educational and sports environments. Analyzing this phenomenon in contexts where children meet and interact, like football schools/clubs, is critical. The study aims to investigate how youth football coaches perceive bullying and their role in addressing it, as well as to explore bullying as a social phenomenon through the lens of coaches' knowledge and experiences.

**Methods:**

Twenty-four coaches from the Portuguese region of Tâmega and Sousa highlighted their awareness and concern about the growth of bullying in football, and that everyone is involved (70.8%).

**Results:**

The victims are “younger” (83.3%) and “teammates” (54.2%) of the aggressors. Assaults occur mostly due to the victims' “physical characteristics”, “behaviors/attitudes” and “sexual orientation”. Coaches believe that the aggressors are “opposing team fans” (25.5%), “teammates” (22.6%), “male” (66.67%), “older” than the victims (75%), and attack in “locker rooms” (23.81%) and in “stands” (17.46%). They consider that bullying victims do not seek help (91.67%) due to “fear” (79.17%), and that the sports agents react indifferently (50%) to acts of this nature.

**Conclusions:**

Coaches acknowledge that they can play a decisive role in preventing this issue, but do not feel fully prepared to identify warning signs and act in accordance, emphasizing the need to improve coach education through new prevention strategies.

## 1 Introduction

The 21st century has witnessed the persistence of a serious problem in multiple contexts, called bullying. This timeless phenomenon has diverse causes and several short, medium, and long-term consequences, leaving scars on various human development levels, such as childhood, adolescence, and even adulthood (1). In literature, this concept is addressed by different authors as a problem that has been assuming increasingly larger dimensions. Bullying derived from the word bully (e.g., oppressor, tyrant) and it denotes aggressive, antisocial, repetitive, and intentional behaviors practiced by one or more individuals (2). Olweus' pioneering studies on bullying provide a foundational framework for understanding why the term bullying is distinct from broader terms like abuse (1, 3). While both involve harm, bullying refers specifically to a repeated, intentional pattern of aggressive behavior that occurs within a power imbalance, often among peers, particularly in school or workplace settings. Otherwise, abuse is defined as a broader term that encompasses a variety of harmful behaviors, including physical, emotional, or sexual harm, that may occur in any relational context (e.g., familial, intimate, institutional) (1, 2).

Bullying is a form of violence and physical or psychological torture to which a stronger peer or group of children subjects a more vulnerable peer, continuously, and unable to defend themself(ves). Thus, this phenomenon entails aggressive, antisocial, and recurrent behaviors that occur among students in the school context (4), characterized by offensive attitudes, intimidation, humiliation, embarrassment, isolation, exclusion, defamation, physical and/or verbal aggression, theft, and it is present in schools, and other educational contexts, although many of these episodes are not openly recognized (5). In Portugal, there is no exact word that translates literally the original meaning of the term bullying, and therefore the English word has been adopted, and research has sought to give great prominence to this phenomenon (6). The main concept of bullying can be divided into various types: verbal, physical, sexual, psychological, social, and property attack, which consists of tampering with the victim's property without their authorization, deliberately damaging it, or stealing it (6, 7). Smith (7) adds the existence of other forms of bullying directed at groups that stand out for some identity aspect, such as racist bullying, bullying based on religious beliefs, and homophobic bullying. Furthermore, Gil-Villa (8) also distinguishes cyberbullying, carried out through new technologies (e.g., mobile phones, the internet, social networks).

In this regard, Fernandes and Seixas (9) states that for bullying is needed the active involvement of at least two subjects, one aggressor and one victim, which can be, in both cases, an individual or group of individuals (10). In the school and/or sports context, the same individual can play the role of victim, aggressor, and observer/witness (9, 10). Additionally, and according to Vaz (6), these aggressive behaviors can be practiced and witnessed in different school and/or sports settings, due to the concentration of many children and adolescents, the scant adult supervision, and the absence of stimulating activities (11, 12). Playgrounds, corridors, bathrooms/locker rooms, school/club entrances and exits are the places where the phenomenon occurs most frequently (11, 13–17), through various forms (e.g., verbal insults, offensive drawings, threats and physical assaults, social exclusion, intimidation, etc.) (18). For example, in these educational places, adolescents with short stature or overweight tend to be seen as unathletic and clumsy, and thus the (lack of) skills and physical appearance become contributing factors to being bullied (11). However, it is known that there are multiple other triggering factors for aggressive behaviors, which if not actively monitored, prevented, and/or intervened, and can turn into traumatic episodes for children and adolescents.

The augmented concern about the increasing bullying forced research to be developed in sports contexts similar to the school environment (13, 17). Sports practice, often seen as an ally in promoting active and healthy lifestyles and combating bullying, can enhance the occurrence of this phenomenon, due to the ingrained culture and the competitive nature, rigorous selection processes, and rules and procedures not always transparent (13). Considering that competition, aggressiveness, and the relentless pursuit of excellence are part of the game, the analysis of the phenomenon of bullying has emerged as a worrying challenge that requires specific analysis and urgency in concrete actions (11).

In the sports setting, and specifically in football youth levels, this phenomenon has been manifesting itself in various ways, affecting not only sports performance but also the physical and emotional well-being of young athletes. Despite sports practice contributing to healthy and active living, through psychomotor development, general physical fitness, social and psychological well-being, cooperation, team spirit, self-confidence, resilience, fair play, and ethical principles in sports (and in life) (19, 49), it can increase, on the other hand, the risk of the children/adolescents being bullied or becoming the aggressor (11, 17). Therefore, sports youth levels should represent not only a training ground for physical, technical, and tactical skills but also a fertile ground for the transmission of fundamental values and ethical principles, such as sportsmanship, honesty, loyalty, attitude correction, acceptance, and mutual respect, as well as respect for the rules of civic and sports conduct. However, this does not always happen, with the normalization of negative and abusive behaviors and the forgetting of these values (20–22, 50).

The literature identifies several risk factors associated with the likelihood of a young athlete becoming a victim of bullying in the sports context, such as morphology and appearance, disability, belonging to ethnic minorities, sexual orientation, and physical and sporting (dis)abilities (16, 23–29). Bullying victims are often those who stand out for being different from their peers. If the “normal” standard implies the ability to play football or other specific sports, then those who cannot or do not wish to participate in these activities may be targeted for aggression. More skilled boys, who typically dominate games, end up marginalizing other colleagues, such as girls and younger children, as there is no protection or encouragement for less physical or particularly demanding activities (30). On the other hand, these boys exhibit higher levels of aggressiveness but not victimization (13, 31). In a study with young football and judo athletes, Escury and Dudink (11) found that 6% of children reported being victims of bullying during sports activities, two to three times a week, compared to the 20% reported daily at school.

Bullying in football youth levels has a negative emotional impact on victims, leading to a possible aversion to sports practice, low self-esteem, isolation, poor academic and athletic performance, and may result in changing clubs or, in extreme cases, dropping out sports altogether (17, 28, 32, 33). This phenomenon not only compromises personal and athletic development but also undermines the foundations of what should be a journey of growth, learning, and comprehensive formation for young football athletes. According to Marivoet (34), violence and unethical behaviors extend not only to football but to all sports, from high-level competition to the lowest levels of participation, requiring accountability and ethical reflection of the ideals underlying sports action at the initial training levels. The role of coaches in sports extends beyond technical instruction to include fostering motivation, character development, and preventing negative behaviors such as bullying. Recent studies indicate that coaches' autonomy-supportive behaviors positively influence the coach–athlete relationship and team efficacy, which in turn reduces athlete burnout (35). Additionally, supportive coaching behaviors are linked to enhanced team cohesion and psychological well-being among athletes (36). Conversely, controlling coaching behaviors can increase athletes' anxiety levels, negatively impacting their performance and mental health (37). Therefore, coaches who adopt positive leadership styles and establish clear behavioral expectations not only enhance athletic performance but also contribute to the psychological well-being of their athletes, reducing the likelihood of harmful dynamics such as bullying.

While school and club environments both play significant roles in youth sports, they differ markedly in structure, objectives, and social dynamics, which can influence athlete experiences and developmental outcomes (38, 39). In this sense, several studies emphasized the importance of understanding and addressing bullying in youth football to develop effective prevention and intervention strategies that are crosscutting to other sports and setting (15, 18). Sports will only play an educational and protective effect if it incorporates a strong pedagogical component that creates a stimulating learning environment, where coaches will play an essential role, creating positive environments and empowering the most vulnerable athletes (22, 27). The study aims to investigate how youth football coaches perceive bullying and their role in addressing it, as well as to explore bullying as a social phenomenon through the lens of coaches' knowledge and experiences.

## 2 Materials and methods

### 2.1 Participants

An exploratory case study of both quantitative and qualitative nature was conducted, through the application of a questionnaire survey. This study involved the participation of twenty-four (24) Portuguese football coaches of both genders (*N* = 23 ♂ and 1 ♀), aged between 20 and 63 years old (*M* = 38.67; SD = 10.85), from various cities in the Portuguese region of Tâmega and Sousa: Amarante, Celorico de Basto, Felgueiras, Lousada, Marco de Canaveses, Paços de Ferreira, Paredes, and Penafiel. Although it was a random sample, it is a male dominated perspective with only one female participant. In fact, the Portuguese coaching context continues to be dominated by male coaches according to a portrait based on statistical indicators collected in the process of issuing Professional Titles between 2010 and 2017 by the Portuguese Institute of Sport and Youth (IDPJ) (40). Also, a limited number of coaches responded to the survey, which may influence the generalizability of the findings. It is possible that respondents were more engaged with the topic of bullying compared to non-respondents, which may have influenced the findings.

Most participants (87.5%) possessed a high level of sports training in football accredited by the Fédération Internationale de Football Association (FIFA) and by the Portuguese Football Federation (FPF)—1st, 2nd, or 4th level of certification (*N* = 21). Eighteen (*N* = 18) coaches reported being former football athletes with an average experience of 10.05 years of practice (SD = 8.10), three (3) coaches were athletes from other sports, and another three (*N* = 3) coaches claimed to have no experience as athletes. Additionally, the participants had diverse academic backgrounds: secondary education (*N* = 11), higher professional technical course (*N* = 1), bachelor's degree (*N* = 10), master's degree (*N* = 1), and doctorate (*N* = 1). Furthermore, the coaches had an average experience as football coaches of 9.46 years (SD = 9.18), and 83.3% emphasized that in the last five years they had coached football teams in youth categories (under-17) in the above-mentioned cities. All ethical guidelines from the Ethics Council of the Higher Institute of Educational Sciences of Douro were considered in data collection and processing (ethical code: CV.ESD.23). The present research was conducted according to the ethical standards of the Declaration of Helsinki. All participants were notified about the aims and risks of the investigation. The study includes only players that have signed the informed consent (40). The questionnaire survey used was composed of three parts dedicated to: (i) sociodemographic data, (ii) coaches' perspectives on the bullying phenomenon, and (iii) respondents' opinions on bullying in youth levels football, its prevention and intervention strategies. Bullying was defined in the survey as repeated, intentional harmful behavior involving a power imbalance, and participants indicated their familiarity with this concept using descriptors such as “well” or “very well”. Also, fear was defined as retaliation, professional repercussions, or exacerbation of conflict if bullying incidents were reported. Finally, the collected data was analyzed considering descriptive and inferential statistics, univariate analyses, and subsequently reviewed and discussed by all the involved researchers.

### 2.2 Data collection

For the questionnaire application, it was necessary, firstly, to promote and disseminate the study in the official organizations, sending emails to the Portuguese Football Federation (FPF), Porto Football Association (AFP), National Association of Football Coaches (ANTF), and their respective associates, managers, and coaches. Participants worked in diverse coaching contexts, including youth sports, elite training, and recreational settings, which may have influenced their experiences and perceptions of bullying. Prospective participants were contacted through the organization's internal mailing list, which included all registered coaches. An email invitation was sent with detailed information about the study's aims, confidentiality assurances, and a link to the online questionnaire, in Google Forms format. Then, this online questionnaire was sent to all football clubs included in the FPF's mailing listing and shared on social media platforms like Facebook, Instagram, and Whatsapp to reach as many coaches from the Tâmega and Sousa region as possible. For data collection, the questionnaire survey was sent to football coaches and Physical Education teachers from the region. The questionnaire included items addressing coaches' familiarity with bullying, its definition, and the types of bullying observed, such as physical, psychological, verbal, and digital forms. Coaches were also asked about the roles of victims and aggressors, the contexts of bullying, and risk factors like physical characteristics, behaviors, and sexual orientation. Additional questions focused on the gender and age of both victims and aggressors, as well as the coaches' training and preparedness to handle bullying incidents. Also, participants were asked to define bullying by selecting from multiple-choice options: (1) repeated, intentional harm directed at an individual; (2) isolated, unintentional harm; (3) competitive behavior among athletes; (4) verbal disputes between peers. This survey was available for response between June 2023 and August 2023 (15, 17, 27, 33). All participants were provided with information regarding the study's aims beforehand, and they were assured of confidentiality in data handling and anonymity throughout the analysis process.

### 2.3 Data analysis

To confirm that the variables were distributed normally, an exploratory study was conducted. Both absolute and relative frequency were used to express the qualitative variable data. For the tests that were conducted, the significance value used to ascertain if there were statistically significant differences was *p* < 0.05. When investigating the relationship between the independent factors under investigation and the existence or lack of a risk of acquiring type 2 diabetes, the chi-square (*χ*^2^) test or Fisher's exact test was utilized. The Cramer's *V* test was also used to determine how strongly were the effects size. The analysis of adjusted residues was carried out in order to comprehend the significance of the variable relationship. Data processing and analysis were conducted using IMB SPSS 26 (Armonk, NY: IBM Corp).

## 3 Results

**Figure 1:**
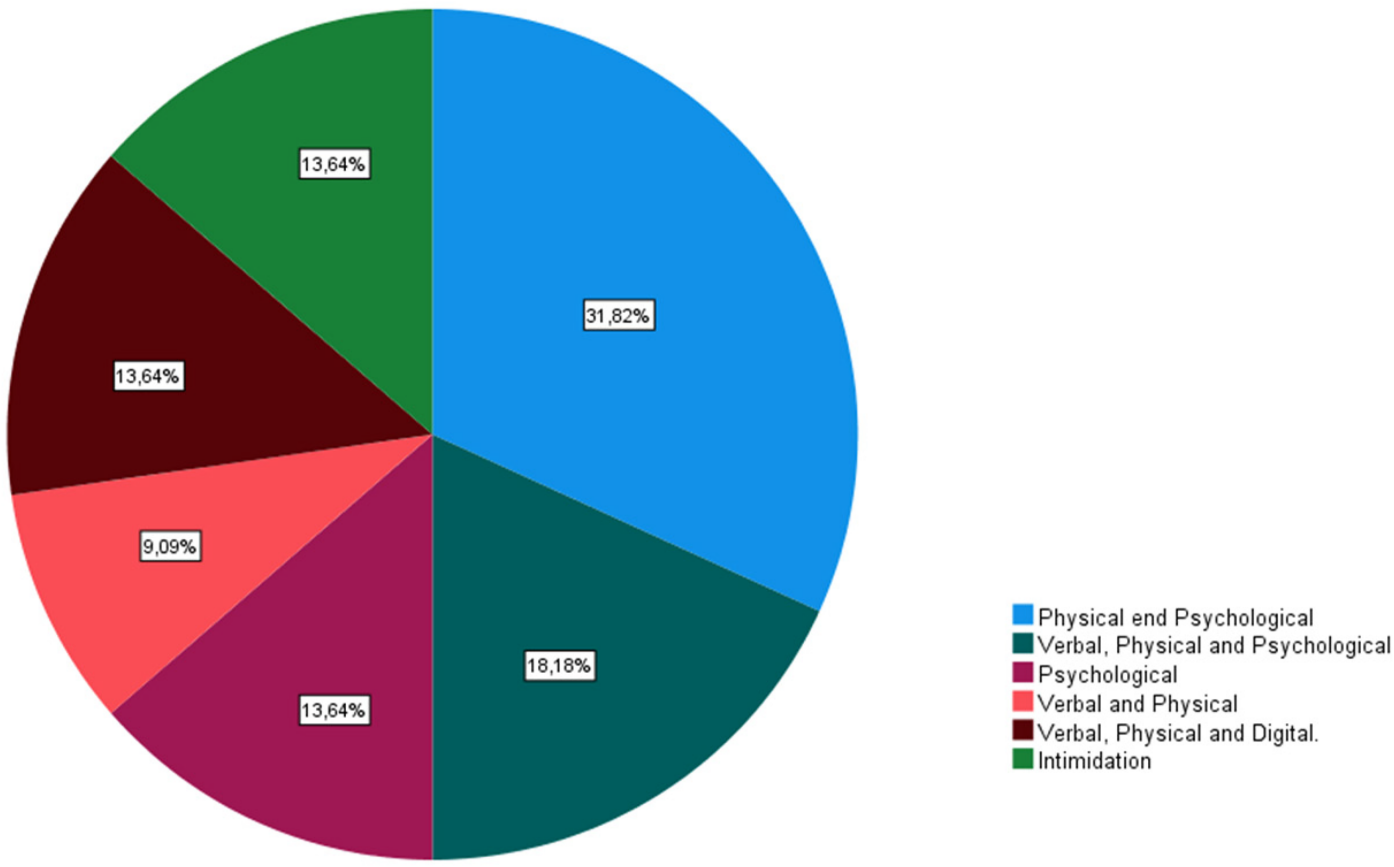
Types of bullying.

The results indicate a broad awareness of bullying among youth football coaches. All surveyed coaches familiar with the phenomenon, with the majority (*N* = 20) stating they knew “well” or “very well” about it. Eleven coaches reported having academic training in the area, and 17 recognized bullying as a problem in sports, particularly in youth football (*N* = 18). When asked about the meaning of bullying, most coaches (83.3%) provided a decisive definition, describing it as repeated, intentional harm directed at an individual. Figure 1 showed the prevalence of type of bullying in this population: physical and psychological (31.82%), verbal, physical and psychological (18.18%), psychological (13.64%), verbal and physical (9.09%), verbal, physical and digital (13.64%), intimidation (13.64%). Delving into this concept, the coaches revealed that they were aware of the existence of different types of bullying (Figure 1). Additionally, the coaches highlighted that bullying victims are from the same team as the aggressors (54.2%), members of another club (29.2%), and/or from the same club (16.7%). According to the respondents, there are several reasons or risk factors that expose athletes to aggression, namely, the physical characteristics of the athlete (*N* = 17), behaviors/actions (*N* = 15), sexual orientation (*N* = 8), sporting performance (*N* = 8), social background (*N* = 4), club (*N* = 3), ethnicity (*N* = 3), and being a girl (*N* = 1).

**Figure 2:**
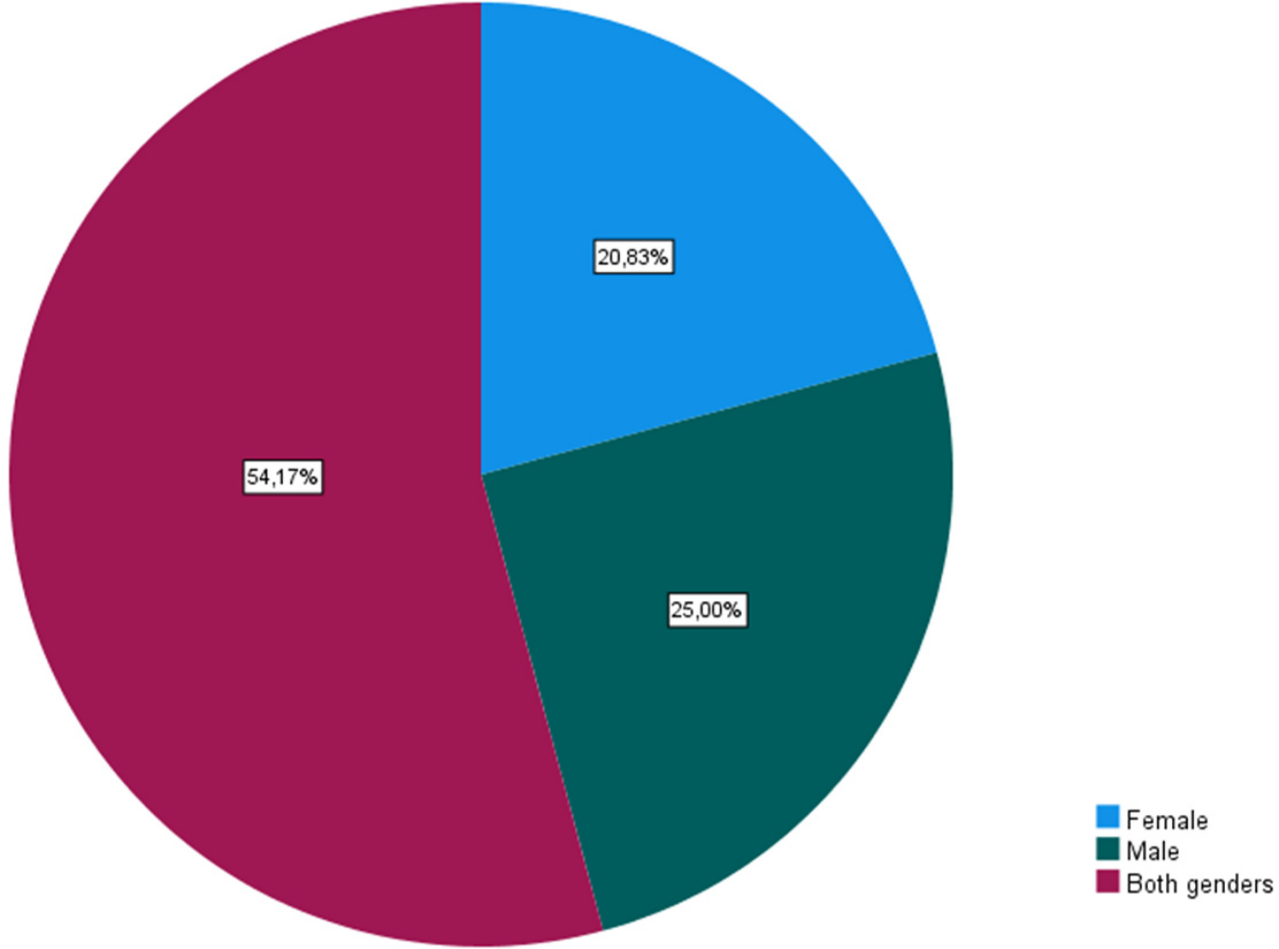
Gender of athletes’ victims of bullying.

Additionally, 70.8% of coaches identified “everyone” as potential participants in bullying, while 20.8% mentioned “aggressors” and 8.3% identified “victims.” When specifying the main victims, 54.2% of coaches cited “teammates”, 15.1% mentioned “referees”, and 5.7% indicated “opposing team players”, with younger athletes (83.3%) being more susceptible to bullying episodes. Coaches highlighted various reasons or risk factors for victimization, with physical characteristics (*N* = 17), behaviors/actions (*N* = 15), and sexual orientation (*N* = 8) being the most common. On the other hand, “opposing team supporters” (25.5%) and “teammates” (22.6%) were identified as the main aggressors. The majority of coaches (75%) attributed the role of aggressor to “older athletes”, followed by both age groups (16.7%), with younger athletes being identified less frequently (8.3%). Figure 2 showed the Gender of athletes' victims of bullying, however no significant differences were found between genders [*χ*^2^(5) = 2.25; *V* = 0.32; *p* = 0.814] (Figure 2).

**Figure 3:**
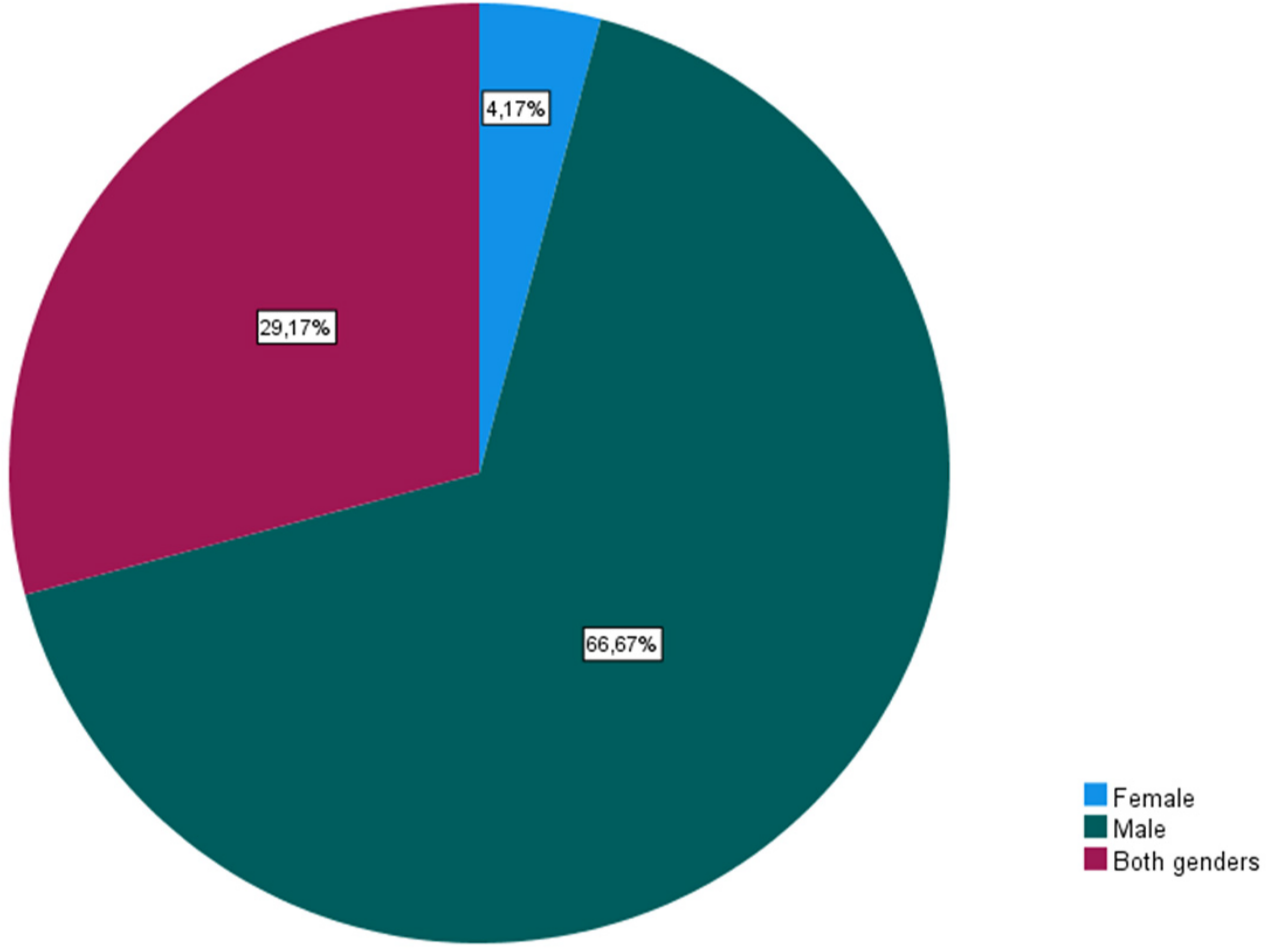
Gender of athletes’ aggressors.

**Figure 4:**
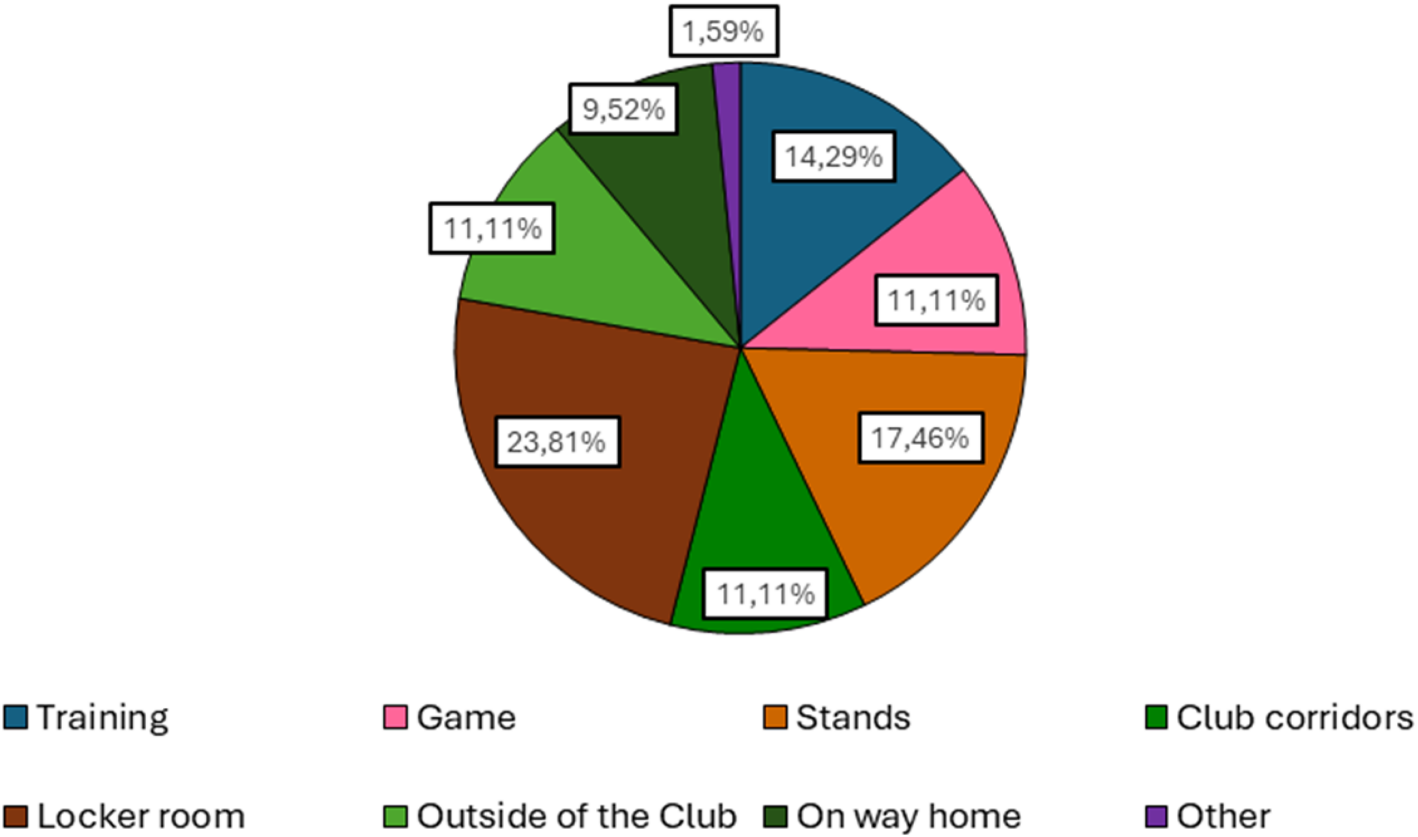
Locations where bullying incidents occur.

**Figure 5:**
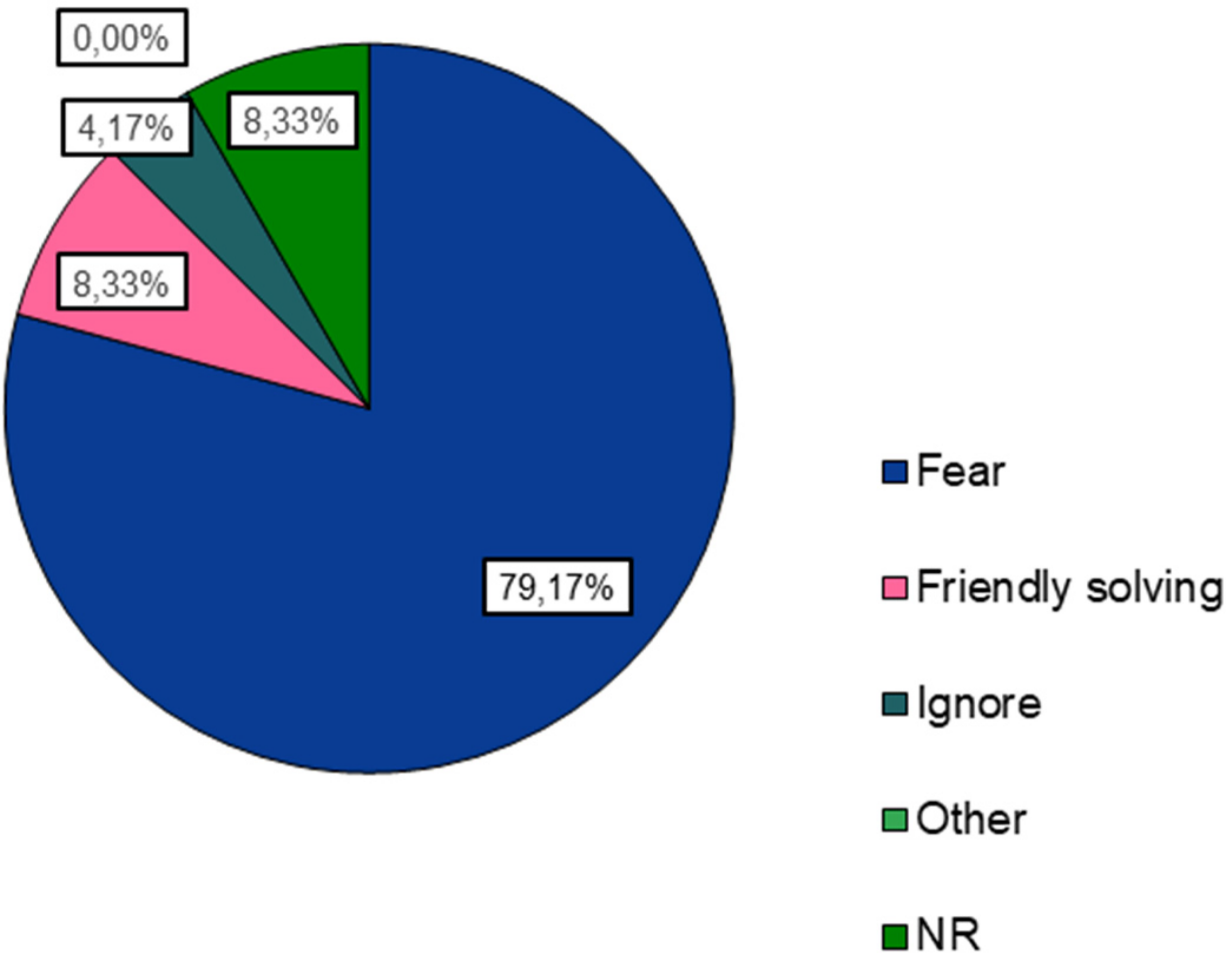
Reasons why bullying victims do not seek help.

**Figure 6:**
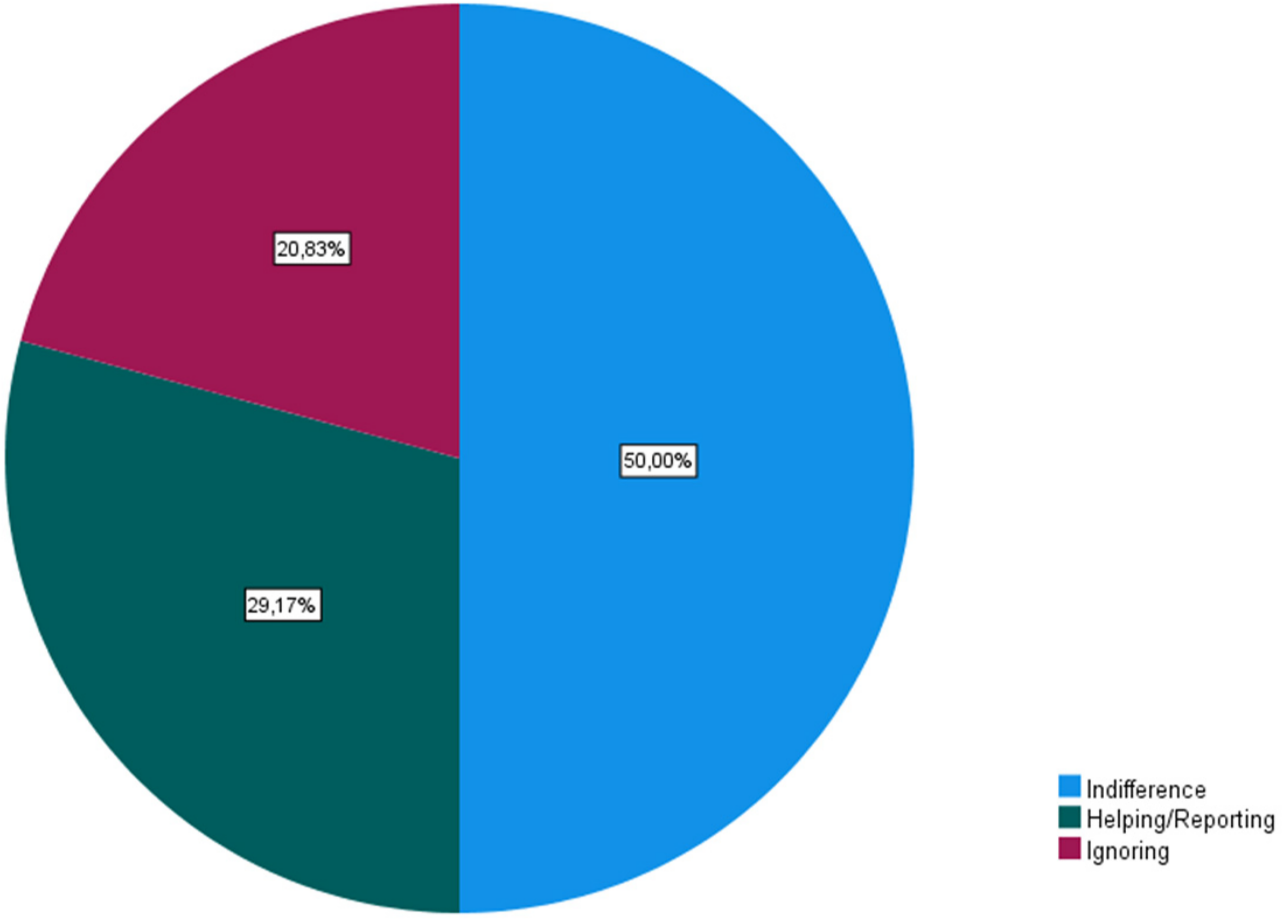
Sports agents’ reactions to bullying episodes.

Finally, 45.8% of coaches believed that the aggressors were from the same team as the victims. While incidents of bullying were reported within clubs, particularly in locker rooms (45.8%), coaches noted that only a small percentage of athletes (8.33%) sought help, with “fear” being the predominant reason for not doing so. According to the respondents, there are several reasons or risk factors that expose athletes to aggression, namely, the physical characteristics of the athlete (*N* = 17), behaviors/actions (*N* = 15), sexual orientation (*N* = 8), sporting performance (*N* = 8), social background (*N* = 4), club (*N* = 3), ethnicity (*N* = 3), and being a girl (*N* = 1). Additionally, eleven (11) coaches disclosed having academic training in this area and identified bullying as a problem in sports in general (*N* = 17) and more specifically in youth football (*N* = 18). Despite recognizing their role in prevention, nearly 30% of coaches felt unprepared to identify warning signs, highlighting the need for more formal education on bullying prevention. In terms of the aggressors' gender, coaches gave boys the most weight, followed by both sexes, and then females (Figures 3–6).

## 4 Discussion

The study aims to investigate how youth football coaches perceive bullying and their role in addressing it, as well as to explore bullying as a social phenomenon from the Portuguese region of Tâmega and Sousa. The study's findings helped to characterize bullying in the Tâmega and Sousa sub-region's youth football development levels, which allowed for a better understanding of coaches’ perceptions and experiences in these levels. Additionally, the study added to the scant literature on bullying in this context, where the previous published studies have concentrated more on victim perception than on prevention and intervention. Coaches are aware of the evolution of bullying in this sporting context and competition levels, and this study has shown that they are concerned about it. They also believe that all sports agents are participating in this phenomenon, albeit they are unsure of exactly who “everyone” is. The findings suggest that while coaches are aware of bullying and its implications, many recognize gaps in their ability to address it effectively, pointing to the need for further training and resources to empower them in their preventive roles.

The existence of bullying in youth sports is well-known (16, 17, 29), yet there is a lack of literature that examines the experiences of how youth football coaches perceive this phenomenon. The statement emphasizes that all surveyed coaches were aware of the concept of bullying, especially within the context of football, and most of them reported being familiar with it to a significant degree (“well” or “very well”). This finding aligns with existing studies that similarly highlight a high level of awareness among sports professionals regarding bullying. For example, research has shown that coaches often recognize bullying as a relevant issue in sports environments, given its prevalence and impact on athletes' well-being. This widespread awareness is likely influenced by increased attention to bullying in both academic literature and public discourse, particularly in youth sports settings (20, 22). When asked about the meaning of the concept of bullying, the coaches (83.3%) were decisive in their definition, stating that “it is a form of repeated, persistent behavior directed at an individual and aimed at causing fear and suffering, harming the body, feelings, self-esteem, or reputation of another person”, which aligns with Olweus (1, 3) findings. Delving into this concept, the coaches revealed that they were aware of the existence of different types of bullying, as observed in some studies (41). Jokes, comments and shouts from the grandstand are usually considered incivility or inappropriate behavior rather than bullying, as they do not always meet the criteria of repetition, intentionality, and a power imbalance (20, 22). However, if such actions are sustained, targeted, and directed at specific individuals, they could be classified as bullying, emphasizing the need to carefully define its boundaries within the context.

Regarding the participants in the bullying phenomenon, the coaches mentioned that the main actors are “everyone” (70.8%), the “aggressors” (20.8%), and the “victims” (8.3%), corroborating Fernandes and Seixas (9). No emphasis was given to the “observers”, in contrast to Lago and colleagues which highlighted their existence and presence in this kind of episode (14). According to the respondents' answers, the main victims of bullying are the “teammates” (54.2%), the “referees” (15.1%), and the “opposing team players” (5.7%), with younger athletes (83.3%) being more susceptible to these episodes, corroborating Higgins (30), followed by older athletes (8.3%). The gender of the athletes was not considered a discriminatory factor for being a victim of bullying, although Evans et al. (13) and Vveinhardt & Fominiene (31). consider this an important factor, even though boys exhibit higher rates of aggression rather than victimization.

Additionally, the coaches highlighted that bullying victims are from the same team as the aggressors (54.2%), members of another club (29.2%), and/or from the same club (16.7%), which is in line with various studies (16, 23, 24, 28, 31, 42, 43). On the other hand, the main aggressors identified by the coaches were “opposing team supporters” (25.5%), “teammates” (22.6%), “parents” (12.5%), and “opposing team players” (12.5%). Coaches mainly attributed the role of aggressor to “older athletes” (75%), followed by athletes from both age groups (16.7%), and lastly to “younger ones” (8.3%). These results differ from those of Nery (44), who states that defining the profile of the aggressor is difficult due to the interaction of behavior and environment. Regarding the gender of the aggressors, coaches placed great emphasis on boys, followed by both genders, and finally girls, corroborating the results found by Vveinhardt & Fominiene (31).

We can also note that 45.8% of the coaches believed that the aggressors are from the same team as the bullying victims, 25% considered them to be members of the same club, 20.8% said that the aggressors are from another club, and 8.3% attributed this role to members of another team. It is difficult to relate these findings to other studies as, to our knowledge, there are no studies that have used the same categorical analysis, specifically categorizing the main victims and aggressors of bullying in sports as strictly teammates, referees, and players from the opposing team, based on the age of the athletes. However, Holt, Finkelhor and Kantor (45) suggested that it may be common for teammates to be perpetrators of bullying, and others consider that referees or even players from the opposing team may be involved, making it difficult to generalize or categorically assign fixed roles of victims and aggressors in that context. Furthermore, the relationship between the age of athletes and susceptibility to bullying episodes may be more complex than a simple hierarchy based on age. While younger athletes may be considered more susceptible due to vulnerability associated with inexperience or immaturity, older athletes may face different forms of pressure or bullying related to performance expectations or social hierarchies within the team (15, 46).

After identifying the main actors in this phenomenon, coaches were asked if they were aware of incidents of bullying in their club(s), and 45.8% of coaches responded affirmatively, pointing to various locations for these occurrences, as shown in Figure 4. The locker room was the most representative, corroborating the results obtained by Escury and Dudink (11), Jachyna (12), and Ríos et al. (47). Coaches were also asked whether they believed young athletes sought help when they were subjected to aggression, and only 8.33% of coaches believed they did, compared to 91.67% who firmly stated that athletes do not seek support, which was also verified in the results obtained by Prat Grau et al. (22). The reasons cited by coaches for athletes not seeking this help were varied, but “fear” was predominant compared to other reasons.

Coaches noted that when faced with an episode of aggression, various sports agents have different reactions, with “indifference” being the most mentioned, followed by “helping the victim/Reporting”, and finally “ignoring” the situation (Figure 6). These data are supported by the study of Escury and Dudink (11). Finally, we can conclude that coaches consider themselves to have a significant role in preventing this issue, which is in line with Baiocco and colleagues (24), but 29.2% of the coaches stated that they do not feel prepared to identify warning signs for several reasons, for example: not having specific training in the area, difficulty in recognizing the signs, not knowing how to act in these situations, and ultimately downplaying them, as corroborated by the findings of Prat Grau et al. (22). However, this is a “phenomenon increasingly visible in football clubs, but in terms of prevention, there is still much to be done”, and it is therefore crucial to consider the formal education on bullying in sports coaching courses, as well as the use of strategies that coaches should be aware of how to implement (22, 48). Bullying in football development levels in the 21st century is a serious concern, and the competitive pressures, pursuit of excellence, and highly aggressive nature of sports and football in particular can create environments prone to this phenomenon, and young athletes can easily become targets of bullying for various reasons (e.g., inferior performance, physical characteristics, different skills, or simply being considered “different” from the group) (11, 12).

The occurrence of this phenomenon can severely impact self-esteem, psychological well-being, and the overall development of young athletes both on and off the field. Although bullying is an increasingly present and visible phenomenon in football clubs, coaches do not feel fully prepared to identify warning signs and act accordingly, despite recognizing that they can play a decisive role in preventing this issue (19, 49). We believe in the pedagogical and formative power of coaches, not only teaching sports skills but also modelling positive behaviors and actively intervening when inappropriate behaviors are observed. Emphasizing personal development and teamwork over just sports results may also help reduce the pressure that contributes to the manifestation of this phenomenon (20–22, 50).

Thus, sports organizations should continue to invest in bullying prevention programs and policies from the earliest development levels, through the education and awareness of coaches, athletes, and parents about the importance of mutual respect, equity, and acceptance of diversity, also based on an education in the values and ethical principles of sports (27, 51). Sports, and football in particular, can and should be allies against bullying, through continuous awareness of this problem and the joint efforts of all involved, from athletes, coaches, parents, referees, sports leaders, and the community at large, will be essential to create a safe and positive environment in football development levels (23–25, 28, 29). Bullying in football development levels in the 21st century is a serious concern, and the competitive pressures, pursuit of excellence, and highly aggressive nature of sports and football in particular can create environments prone to this phenomenon, and young athletes can easily become targets of bullying for various reasons (e.g., inferior performance, physical characteristics, different skills, or simply being considered “different” from the group) (17, 29). The occurrence of this phenomenon can severely impact self-esteem, psychological well-being, and the overall development of young athletes both on and off the field. Although bullying is an increasingly present and visible phenomenon in football clubs, coaches do not feel fully prepared to identify warning signs and act accordingly, despite recognizing that they can play a decisive role in preventing this issue (16). We believe in the pedagogical and formative power of coaches, not only teaching sports skills but also modelling positive behaviors and actively intervening when inappropriate behaviors are observed. Emphasizing personal development and teamwork over just sports results may also help reduce the pressure that contributes to the manifestation of this phenomenon (23–25).

Thus, sports organizations should continue to invest in bullying prevention programs and policies from the earliest development levels, through the education and awareness of coaches, athletes, and parents about the importance of mutual respect, equity, and acceptance of diversity, also based on an education in the values and ethical principles of sports (23, 24, 28). Sports, and football in particular, can and should be allies against bullying, through continuous awareness of this problem and the joint efforts of all involved, from athletes, coaches, parents, referees, sports leaders, and the community at large, will be essential to create a safe and positive environment in football development levels (16, 31, 42, 43).

There are limitations in this study that we can report to explain the current research. Another limitation may be related to the use of closed-ended responses in the data collection instrument. Possibly, the use of open-ended responses would provide more detailed content and practical evidence regarding coaches' perceptions and experiences. Lastly, while we consider the sample size reasonable considering the selected geographic sub-region, we highlight as a limitation the small number of participants, which does not allow us to analyze in greater depth or generalize the results to all football clubs and schools in the region. Future research should expand this survey to other areas of the country to carry out a more realistic prospective study. While the exact number of coaches reached is unavailable, the sample size reflects those who were likely more engaged with the topic. Additionally, more studies on bullying in others team sports are necessary to compare contexts and contribute new knowledge. Although some minor clarifications and adjustments are needed to improve clarity, the issues raised are of a secondary nature in youth and professional football in general. More specifically, the perception of female soccer coaches should be applied, given the small number of coaches who participated in this study.

## 5 Conclusions

The results of this study contributed to characterizing bullying in youth football development levels in the Tâmega and Sousa sub-region, enabling a better understanding of coaches' perceptions and experiences in these levels and a contribution to the still limited literature on bullying in this context, where most studies to date have focused on the victim's perception rather than prevention and intervention. Through this study, it became clear that coaches are aware of and concerned about the evolution of bullying in this sport and at these levels, and they believe that all sports agents are involved in this phenomenon, with no clarity on who “everyone” is. For the respondents, the victims of aggression are mainly teammates and usually younger than the aggressors, and they are so due to their physical, behavioral, and performance characteristics. Coaches also understand that these victims do not seek help of fear and because various sports agents react with “indifference” to these episodes. On the other hand, the aggressors are mainly supporters of the opposing team, male, and older than the victims, and they mostly attack in locker rooms and stands.

## Data Availability

The raw data supporting the conclusions of this article will be made available by the authors, without undue reservation.
